# Fracture Resistance of CAD/CAM-Fabricated Zirconia and Lithium Disilicate Crowns with Different Margin Designs: Implications for Digital Dentistry

**DOI:** 10.3390/jfb16060205

**Published:** 2025-06-02

**Authors:** Tareq Hajaj, Diana Marian, Cristian Zaharia, Serban Talpos Niculescu, Radu Marcel Negru, Florina Titihazan, Mihai Rominu, Cosmin Sinescu, Andreea Codruta Novac, Gabriel Dobrota, Ioana Veja

**Affiliations:** 1 Department of Prostheses Technology and Dental Materials, Faculty of Dentistry, Victor Babes University of Medicine and Pharmacy, 2 Eftimie Murgu Sq., 300041 Timisoara, Romania; tareq.hajaj@umft.ro (T.H.); florina.titihazan@umft.ro (F.T.); rominu.mihai@umft.ro (M.R.); sinescu.cosmin@umft.ro (C.S.); cojocariu.andreea@umft.ro (A.C.N.); 2 Research Center in Dental Medicine Using Conventional and Alternative Technologies, Faculty of Dental Medicine, Victor Babes University of Medicine and Pharmacy of Timisoara, 9 Revolutiei 1989 Ave, 300070 Timisoara, Romania; 3 Department of Dental Medicine, Faculty of Dentistry, “Vasile Goldis” Western University of Arad, Str. Liviu Rebreanu 86, 310045 Arad, Romania; marian.diana@uvvg.ro (D.M.); veja.ioana@uvvg.ro (I.V.); 4 Department of Oral and Maxillofacial Surgery, Faculty of Dentistry, Victor Babes University of Medicine and Pharmacy, 2 Eftimie Murgu Sq., 300041 Timisoara, Romania; 5 Department of Mechanics and Strength of Materials, Polytechnic University of Timișoara, 2 Piata Victoriei 2., 300006 Timisoara, Romania; radu.negru@upt.ro; 6 Department of Prosthodontics, Faculty of Dentistry, Victor Babes University of Medicine and Pharmacy, 2 Eftimie Murgu Sq., 300041 Timisoara, Romania; drgabrieldobrota@gmail.com

**Keywords:** zirconia crowns, lithium disilicate, fracture resistance, cervical margin, chamfer, tangential, CAD/CAM, adhesive cementation

## Abstract

Objective: This in vitro study aimed to evaluate the influence of cervical margin design—tangential versus chamfer—on the fracture resistance of monolithic crowns fabricated from lithium disilicate and zirconia ceramics. Materials and Methods: Forty extracted human molars were randomly assigned to two preparation types: chamfer and tangential. Each group was restored with CAD/CAM-fabricated crowns made from either zirconia (IPS e.max^®^ ZirCAD Prime) or lithium disilicate (IPS e.max^®^ CAD), resulting in four subgroups (*n* = 10). Standardized adhesive cementation protocols were applied. After 24 h storage in distilled water, the specimens underwent static load-to-failure testing using a ZwickRoell ProLine Z005 universal testing machine. Results: Zirconia crowns with chamfer margins exhibited the highest mean fracture resistance (2658 N), while lithium disilicate crowns with tangential margins showed the lowest (1862 N). Chamfer preparation significantly increased the fracture resistance of lithium disilicate crowns (*p* < 0.01), whereas margin design had no significant effect on zirconia. All restorations exceeded physiological masticatory forces, confirming their clinical viability. Conclusions: Cervical margin design significantly affected the fracture performance of lithium disilicate crowns but not zirconia. Chamfer preparations are recommended when using lithium disilicate to optimize mechanical strength. These findings underscore the importance of preparation geometry in guiding material selection for CAD/CAM ceramic restorations.

## 1. Introduction

The long-term success of full-coverage crowns in restorative dentistry depends on a combination of factors, including the mechanical properties of the material, the bonding protocol, the digital workflow, and the design of the tooth preparation. Among these, the cervical margin geometry significantly affects both the mechanical behavior of the restoration and its biological integration [1,2,3,4,5]. Current trends favor minimally invasive preparations and CAD/CAM workflows, prompting a clinical need to understand how conservative margin designs interact with different restorative materials under functional loads [6,7].

In recent years, there has been a growing clinical shift toward minimally invasive preparation techniques, including tangential margins, due to their ability to conserve sound tooth tissue and reduce the biological cost of prosthetic treatment [8,9]. At the same time, patient expectations for highly esthetic, metal-free restorations have driven the widespread adoption of advanced ceramic materials and adhesive cementation strategies [10,11]. Yet, clinicians must navigate this evolving landscape with limited guidance on how preparation geometry interacts with different materials under functional conditions. This underscores a critical clinical need: understanding how margin design affects the structural performance of commonly used restorative ceramics [6]. Contemporary all-ceramic materials—including zirconia and lithium disilicate—offer varied balances between strength and esthetics, aligning with evolving demands for minimally invasive, metal-free restorations [10,11,12,13].

Zirconium oxide (zirconia) is a high-strength ceramic widely used for crowns and fixed prostheses due to its superior flexural strength and fracture toughness compared to traditional porcelain [10]. Initially applied in bilayer designs with veneering porcelain for improved esthetics [10,14], these restorations were prone to chipping and delamination due to residual stresses and material mismatches [15]. To overcome this, monolithic zirconia crowns were introduced, eliminating the weak veneer–core interface and providing enhanced structural integrity [15]. With flexural strength ranging from 800 to 1200 MPa, they support minimally invasive preparations and are well suited for posterior and high-load clinical applications, despite their lower translucency [16].

Alongside zirconia, lithium disilicate glass-ceramic has become a cornerstone of all-ceramic restorative systems. Introduced in the late 1990s (e.g., IPS e.max^®^ Empress 2), lithium disilicate combines moderate flexural strength (350–400 MPa) with high translucency, making it ideal for single-unit restorations in esthetically demanding areas [17]. Its microstructure, composed of rod-like Li_2_Si_2_O_5_ crystals embedded in a glassy matrix, allows for monolithic applications or use as a core with layered enamel porcelain. Lithium disilicate’s optical properties closely mimic natural enamel and dentin, delivering superior outcomes in anterior and premolar regions [8]. While lithium disilicate offers high esthetic appeal and clinical success, its lower fracture resistance compared to zirconia makes it more vulnerable in posterior or high-stress situations.

These advancements have significantly expanded the range of metal-free options available to clinicians. The selection between zirconia and lithium disilicate is often dictated by the case requirements: zirconia is preferred for strength-intensive applications or where minimal tooth reduction is necessary, whereas lithium disilicate is favored for its esthetic properties in the anterior region [8]. Ongoing material innovations, such as enhanced translucency in zirconia and reinforced glass-ceramics, continue to refine these options. However, a persistent knowledge gap remains regarding how margin geometry—particularly chamfer versus tangential designs—affects the fracture resistance of these materials.

Zirconia and lithium disilicate remain the most commonly used monolithic ceramics, offering distinct advantages in strength and esthetics, respectively [8,18]. Recent finite element studies and systematic reviews published between 2023 and 2025 have further examined how margin geometry, loading patterns, and material structure influence stress distribution and clinical performance [5,6,17]. However, experimental studies assessing the combined influence of preparation design and material type on fracture resistance are still limited.

In this study, chamfer and tangential (feather-edge) margin designs were selected due to their clinical relevance in contemporary adhesive and CAD/CAM-based restorative workflows. Chamfer margins are widely accepted for all-ceramic crowns, offering a favorable balance between structural support and ease of preparation [1,5,16]. Tangential margins, on the other hand, represent a minimally invasive alternative that preserves tooth structure and is increasingly adopted in biologically oriented preparation techniques (BOPT) [8,17]. While shoulder margins have traditionally been used for metal-ceramic or layered restorations due to their well-defined geometry, they require substantial tooth reduction and are less commonly used in monolithic ceramic workflows [16]. Therefore, shoulder preparations were excluded from this analysis to better reflect current conservative clinical practices and to evaluate the mechanical implications of commonly used margin designs in full-contour CAD/CAM restorations.

Therefore, the present in vitro study aimed to evaluate the influence of cervical margin design—chamfer versus tangential—on the fracture resistance of monolithic crowns fabricated from zirconia and lithium disilicate, using standardized CAD/CAM and adhesive cementation protocols. The null hypothesis (H_0_) was that margin design has no significant effect on the fracture resistance of either ceramic material. The alternative hypothesis (H_1_) posited that margin design does influence fracture resistance, with chamfer preparations yielding higher fracture loads than tangential margins, particularly for lithium disilicate crowns. The findings of this study are intended to help clinicians make evidence-based decisions regarding material selection and preparation design that optimize both mechanical performance and esthetic outcomes.

## 2. Materials and Methods

### 2.1. Study Design and Ethical Approval

This in vitro study was conducted at the Faculty of Dentistry, “Victor Babeș” University of Medicine and Pharmacy of Timișoara, in collaboration with the Department of Materials Resistance at the Polytechnic University of Timișoara. The study protocol received ethical approval from the university’s Ethics Committee (No. 78/08.01.2024, rev. 2025). Informed consent was obtained from all patients prior to tooth donation in accordance with institutional guidelines

### 2.2. Sample Selection and Storage

Forty sound human mandibular molars extracted for periodontal reasons were collected for use in this study. All teeth were cleaned of debris and stored in 0.1% thymol solution at room temperature to prevent dehydration and microbial contamination. The inclusion criteria required structurally intact crowns without caries, restorations, cracks, or signs of demineralization. Teeth were excluded if they exhibited carious lesions, previous restorations, structural loss, or any endodontic intervention.

### 2.3. Tooth Preparation Protocol

Teeth were randomly allocated into four experimental groups (*n* = 10 per group) according to two variables: (1) restorative material (zirconia or lithium disilicate) and (2) cervical margin design (chamfer or tangential). Preparations were performed by a single experienced operator using a high-speed handpiece under continuous water cooling.

For zirconia crowns, axial reduction was approximately 1.0 mm; for lithium disilicate, 1.2–1.5 mm. Chamfer margins were prepared with a uniform depth of 0.8 mm. Tangential preparations followed a tangential approach with no distinct margin design. All internal line angles were rounded to reduce stress concentration.

**Figure 1:**
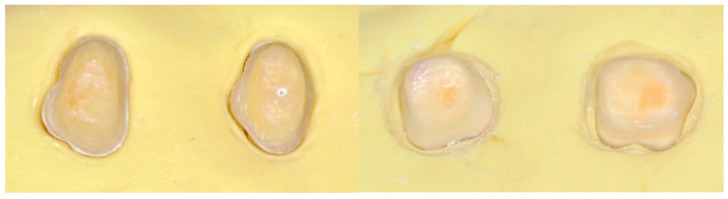
Representative tooth preparations illustrating the two cervical margin designs evaluated in this study: chamfer design (**left**) and tangential design (**right**). These geometries influence the distribution of occlusal stress and may affect the fracture resistance of ceramic restorations.

Each prepared tooth was embedded in self-curing acrylic resin up to 2 mm below the cementoenamel junction (CEJ) to simulate periodontal bone support (Figure 1).

Each prepared tooth was embedded in a self-curing acrylic resin (Technovit^®^ 4004, Kulzer GmbH, Hanau, Germany) up to 2 mm below the cementoenamel junction (CEJ) to simulate alveolar bone support. Although this method is standard in many in vitro studies, it does not replicate the viscoelastic behavior of the periodontal ligament. The absence of a periodontal ligament simulation medium (e.g., polyvinylsiloxane or silicone layer) is a recognized limitation, as it may influence stress distribution and fracture propagation during load application.

After load-to-failure testing, fractured specimens were visually examined under ambient light to characterize the failure mode. Fractures were classified as adhesive, cohesive, or catastrophic. However, no microscopic (e.g., SEM) or stereomicroscopic evaluation was performed, which limits detailed analysis of crack origin and propagation.

### 2.4. Crown Fabrication Workflow

**Figure 2:**
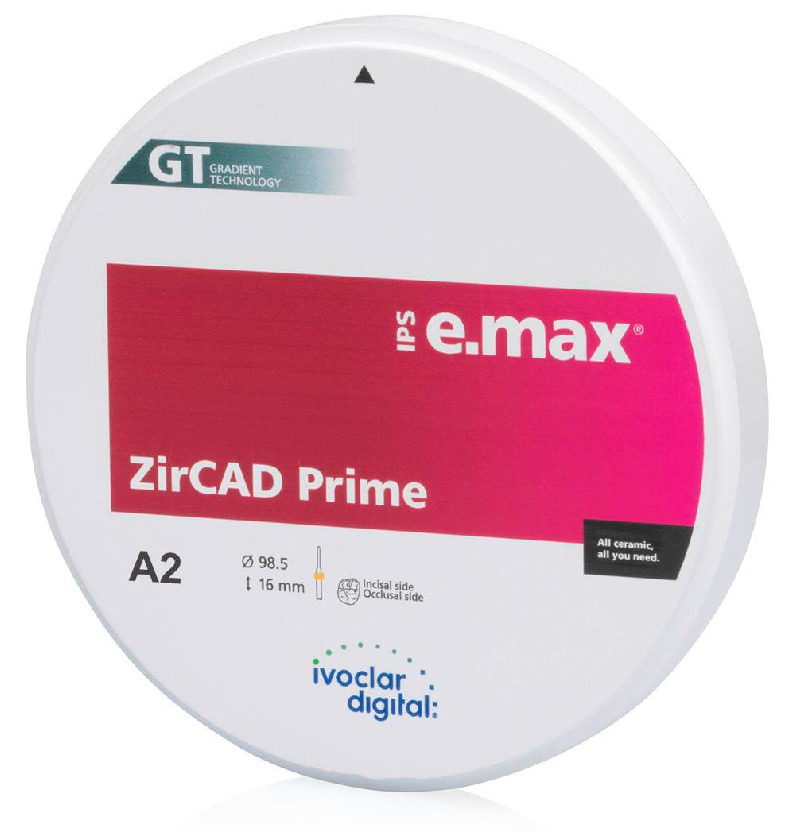
IPS e.max^®^ ZirCAD Prime, Ivoclar Vivadent.

**Figure 3:**
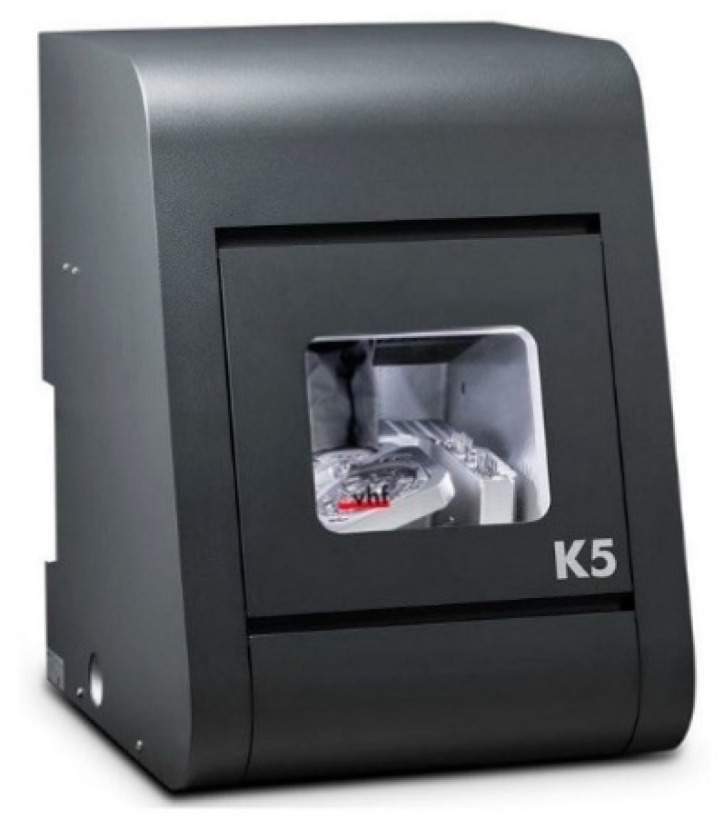
CAD-CAM VHF milling system.

The zirconia crowns were fabricated from IPS e.max^®^ ZirCAD^®^ Prime (Ivoclar Vivadent, Figure 2), a multilayered zirconia that combines 3 mol% and 5 mol% yttria-stabilized zirconia (3Y-TZP and 5Y-TZP) to achieve both high mechanical strength and improved translucency through a gradual gradient in structural and optical properties. Crowns were milled using a CAD/CAM system and sintered at approximately 1500 °C for 2 h, in accordance with the manufacturer’s recommendations (Figure 3).

**Figure 4:**
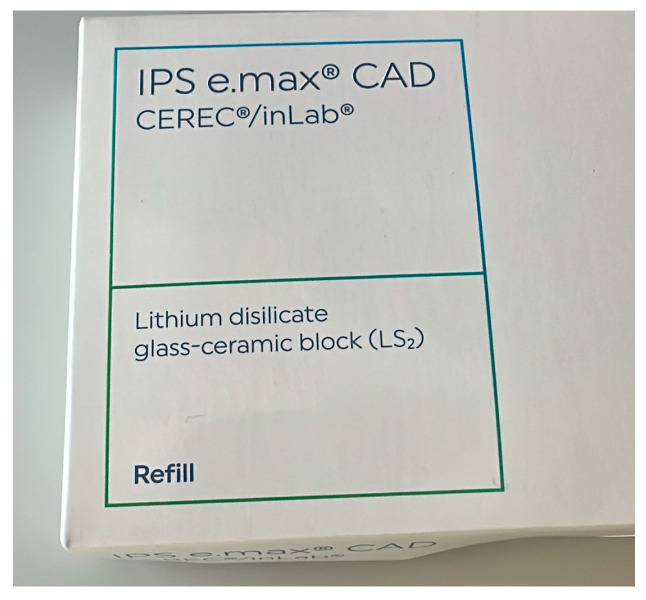
IPS e.max^®^ CAD CEREC, Ivoclar Vivadent.

Lithium disilicate crowns (IPS e.max^®^ CAD, Ivoclar Vivadent; Figure 4) were similarly fabricated using CAD/CAM technology and subjected to a crystallization process according to manufacturer instructions.

All crowns were anatomically contoured and designed with a uniform internal cement space of 50 µm to ensure standardized seating and fit across groups.

### 2.5. Surface Conditioning and Cementation

Zirconia crowns were air-abraded with 50 µm aluminum oxide at 2.5 bar pressure for 10 s from a distance of 10 mm, followed by the application of ScotchBond™ Universal Plus Adhesive (3M ESPE, St. Paul, MN, USA) as a primer.

**Figure 5:**
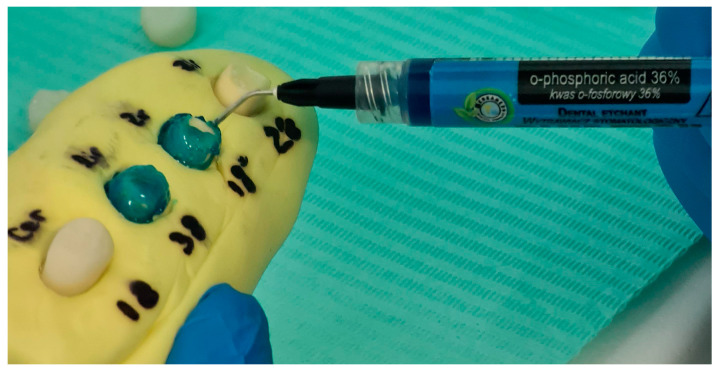
Conditioning of the tooth structure with 36% phosphoric acid (H_3_PO_4_) during adhesive surface pretreatment.

Lithium disilicate crowns were etched with 5% hydrofluoric acid for 20 s, then rinsed, dried, and silanized for 60 s. Tooth surfaces were conditioned with 36% phosphoric acid: 30 s on enamel and 15 s on dentin, followed by rinsing and gentle air-drying to maintain moist dentin (Figure 5).

**Figure 6:**
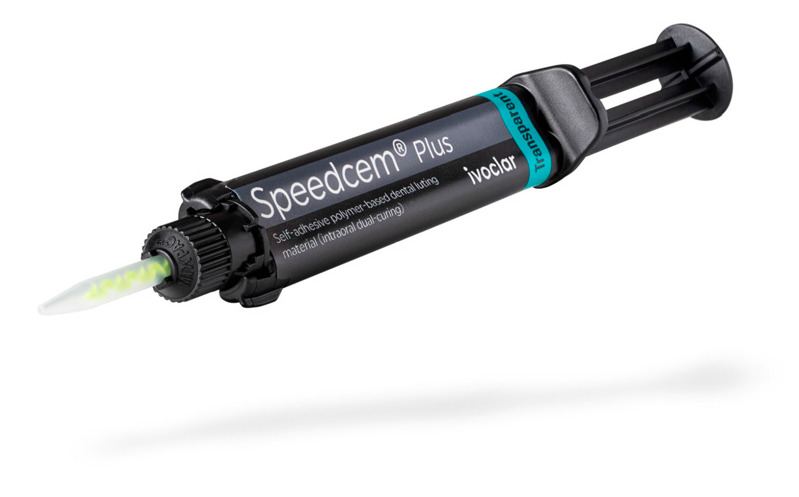
SpeedCEM^®^ Plus (Ivoclar Vivadent), a dual-cure self-adhesive resin cement used for crown luting.

**Figure 7:**
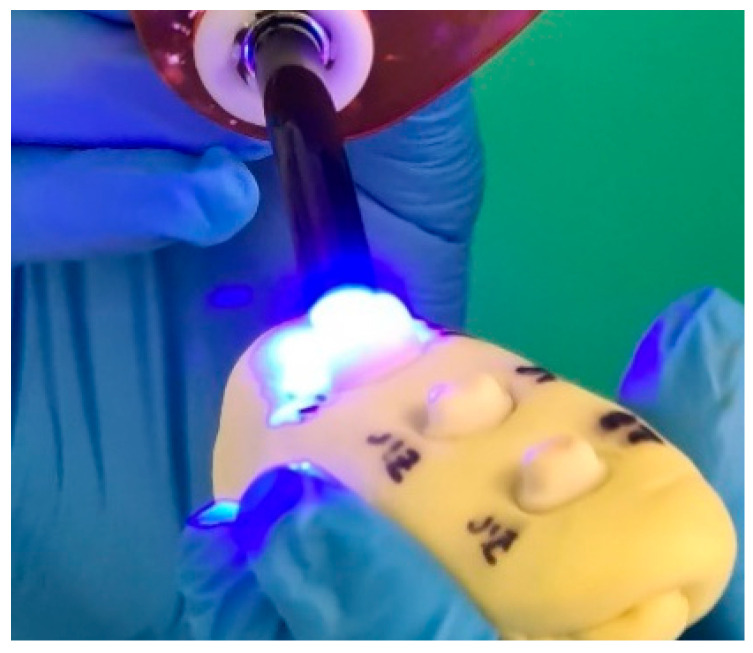
Light-curing of the restorations using a LED polymerization unit (Bluephase N^®^, Ivoclar Vivadent; 800 mW/cm^2^) for 20 s per surface.

Cementation was performed using SpeedCEM^®^ Plus (Ivoclar Vivadent) (Figure 6), a dual-cure self-adhesive resin cement. Crowns were seated with finger pressure, and excess cement was tack-cured and removed, followed by final light curing for 20 s per surface using a LED polymerization light (Bluephase N^®^, Ivoclar Vivadent; 800 mW/cm^2^) Figure 7. All specimens were stored in distilled water at room temperature for 24 h prior to mechanical testing.

### 2.6. Fracture Resistance Testing

**Figure 8:**
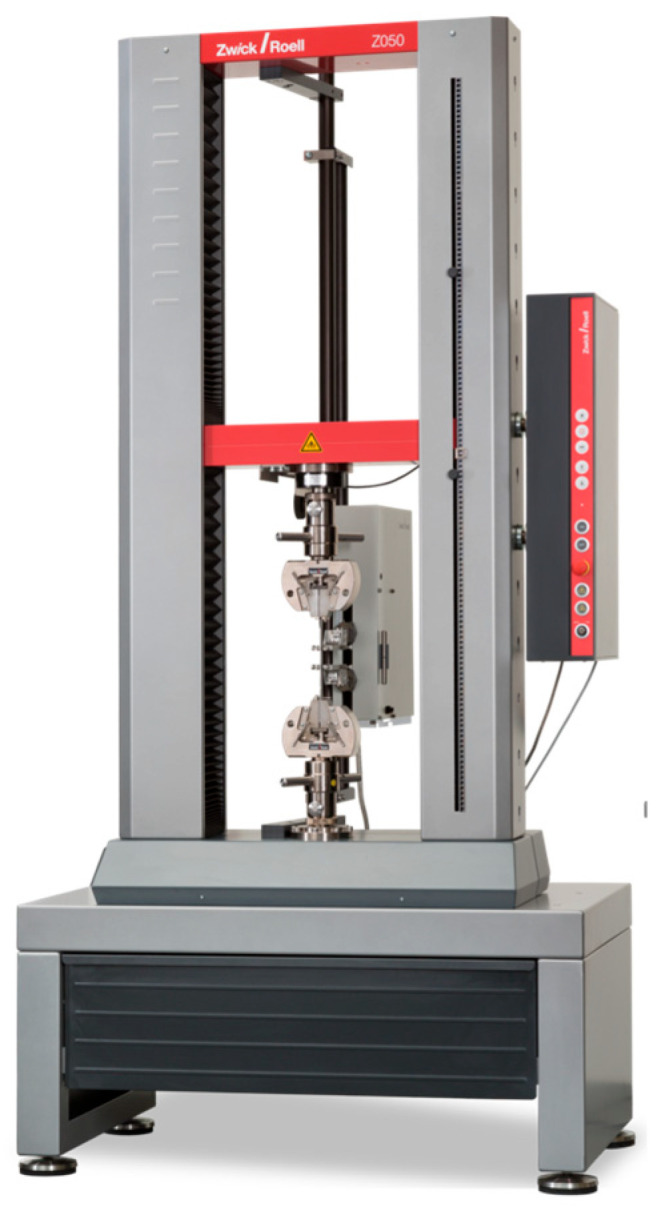
ZwickRoell ProLine Z005.

**Figure 9:**
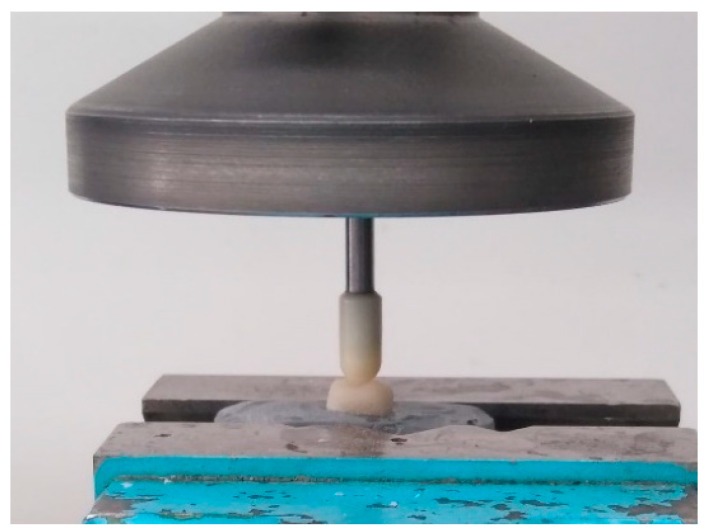
Zirconia rod applying occlusal force.

Load-to-failure testing was conducted using a universal testing machine (ZwickRoell ProLine Z005, ZwickRoell GmbH & Co. KG, Ulm, Germany Figure 8). Each specimen was positioned so that a flat-ended zirconia rod with a 6 mm diameter applied vertical compressive force perpendicular to the occlusal surface at a crosshead speed of 1 mm/min until catastrophic failure occurred (Figure 9). The rod was aligned centrally on the occlusal surface, contacting the central fossa and adjacent cusp slopes, to provide a broad, uniform contact area across multiple inclines. This setup was selected to simulate axial occlusal loading and to ensure standardized stress distribution among all specimens. While commonly used in static fracture testing, this method does not replicate the complex, multi-directional occlusal contacts encountered in vivo.

Fracture loads were recorded in Newtons (N). After testing, fractured specimens were visually inspected under ambient light to determine the mode of failure. Fractures were classified as adhesive (at the interface between crown and cement), cohesive (within the ceramic material), or catastrophic (complete bulk fracture involving both the restoration and underlying tooth structure). No microscopic or SEM analysis was conducted to further evaluate fracture surfaces, which is acknowledged as a limitation of this study.

A flat-ended 6 mm diameter zirconia rod was chosen to apply axial compressive force perpendicular to the occlusal surface. This approach allowed for uniform load application across samples, facilitating standardized comparison. While commonly used in static fracture resistance studies, this method does not replicate the tripodal or cusp-to-fossa loading patterns observed in vivo, which involve complex, oblique contacts. Furthermore, a crosshead speed of 1 mm/min was employed, as recommended by ISO 6872 [17] for ceramic materials testing, providing consistent loading across specimens. Nevertheless, these parameters do not mimic the dynamic nature of mastication, and the results should be interpreted accordingly.

### 2.7. Statistical Analysis

The required sample size was calculated a priori using G*Power (version 3.1.9.7) for a two-way ANOVA with fixed effects, assuming a moderate effect size (f = 0.40), significance level α = 0.05, power (1 − β) = 0.80, and four groups. This analysis indicated a minimum of 7 specimens per group; to enhance statistical robustness and account for potential variability, 10 specimens per group were included. The independent variables were the type of restorative material (zirconia vs. lithium disilicate) and the cervical margin design (chamfer vs. tangential), while the dependent variables were fracture resistance (measured in Newtons) and failure mode (classified as adhesive, cohesive, or catastrophic). Statistical analyses were conducted using MedCalc^®^ version 23.0.6 (MedCalc Software Ltd., Ostend, Belgium). A two-way analysis of variance (ANOVA) was performed to assess the main effects and their interaction. Tukey’s post hoc test was applied for multiple comparisons, with a significance threshold of *p* < 0.05. Assumptions of normality and homogeneity of variance were confirmed using the Shapiro–Wilk test (*p* > 0.05 for all groups) and Levene’s test (*p* = 0.14), respectively. Effect sizes were reported using partial eta squared (η^2^): material type showed a significant main effect (η^2^ = 0.49, *p* < 0.001), and a significant interaction was observed between material and margin design (η^2^ = 0.17, *p* < 0.01). Margin design alone did not reach statistical significance (η^2^ = 0.07, *p* = 0.08). The results are presented as mean ± standard deviation (SD).

## 3. Results

### 3.1. Fracture Resistance and Failure Mode

All crowns exhibited fracture loads substantially exceeding physiological masticatory forces (294–784 N), confirming their mechanical suitability for clinical application.

Most failures were either cohesive—occurring within the ceramic material—or catastrophic, involving complete bulk fracture of both the restoration and underlying tooth structure. No adhesive failures (at the crown–cement interface) were observed in any group. Catastrophic fractures predominated in lithium disilicate crowns with tangential margins, while zirconia crowns primarily exhibited cohesive failure modes.

Fracture mode distribution differed notably between the two materials. In the zirconia groups, 70% of specimens exhibited cohesive fractures and 30% showed catastrophic failure. In contrast, the lithium disilicate groups exhibited 60% catastrophic fractures and 40% cohesive fractures. These distributions are summarized in Table 1.

### 3.2. Influence of Margin Design and Material Type

Mean fracture loads for each group are presented in Table 2. The highest value was recorded in the zirconia–chamfer group (2658 ± 245 N), followed by lithium disilicate–chamfer (2494 ± 205 N). The lowest value occurred in the lithium disilicate–tangential group (1862 ± 230 N).

Within the lithium disilicate group, chamfer margins significantly improved fracture resistance compared to tangential margins (*p* < 0.01). In contrast, zirconia crowns showed no significant difference between chamfer (2658 ± 245 N) and tangential (2425 ± 240 N) preparations (*p* = 0.19), indicating less sensitivity to margin geometry.

### 3.3. Statistical Significance and Interactions Effects

Two-way ANOVA revealed a significant main effect of material on fracture resistance (*p* < 0.001), with zirconia crowns outperforming lithium disilicate overall. The main effect of margin design was not statistically significant when averaged across materials (*p* = 0.08). However, a significant interaction between material and margin design was found (*p* < 0.01), demonstrating that margin configuration influences fracture behavior differently depending on the ceramic used.

**Figure 10:**
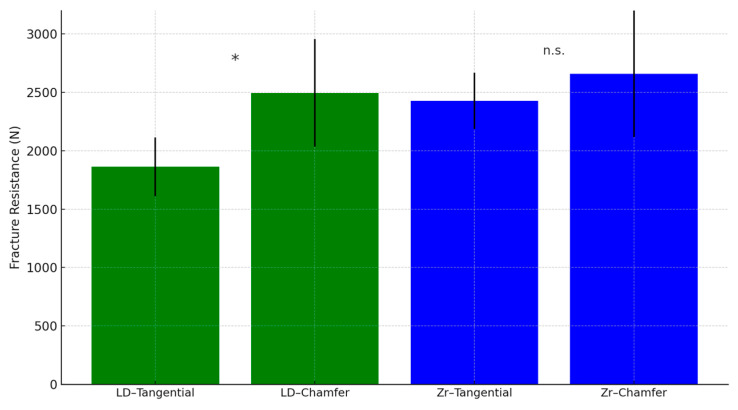
Fracture resistance of monolithic crowns with different margin designs. Bar graph representation of mean fracture resistance (±SD) for each group (*n* = 10). Error bars represent standard deviations. Asterisks (*) indicate statistically significant differences (*p* < 0.01) between margin types within the same material, n.s. indicateno significant difference, as determined by Tukey’s post hoc test.

Post hoc comparisons confirmed that chamfer margins significantly improved fracture resistance only in the lithium disilicate group (*p* < 0.01), but not in zirconia (*p* = 0.19). When comparing materials within the same margin type, zirconia demonstrated superior strength in both tangential (*p* < 0.01) and chamfer configurations (*p* > 0.05, not significant) (Figure 10).

In summary, margin design significantly influenced the fracture resistance of lithium disilicate crowns but had a limited impact on zirconia. All tested crowns withstood forces above the physiological range, supporting their clinical viability. These results underscore zirconia’s superior load-bearing performance and highlight the need for robust cervical design when using glass-ceramics like lithium disilicate.

Mean fracture loads with standard deviations and 95% confidence intervals (CIs) for each group are presented in Table 1. The highest mean value was observed in the zirconia–chamfer group, while the lowest was in the lithium disilicate–tangential group. Despite noticeable variability, all CIs remained above physiological masticatory force thresholds, supporting clinical viability.

Two-way ANOVA revealed a significant interaction between material type and margin design (*p* < 0.01), indicating that the effect of margin geometry on fracture resistance depended on the ceramic used. The main effect of material type was statistically significant (*p* < 0.001), with zirconia showing higher fracture resistance overall. Margin design alone did not reach statistical significance across all groups (*p* = 0.08). The complete statistical summary is presented in Table 3.

## 4. Discussion

This study demonstrated a material-dependent effect of cervical margin design on the fracture resistance of monolithic restoration. Lithium disilicate crowns exhibited significantly lower resistance when fabricated with tangential margins compared to chamfer margins, confirming the mechanical relevance of preparation geometry for glass-ceramics. In contrast, zirconia crowns maintained comparable fracture resistance across both margin types, indicating greater resilience to geometric variation.

These findings suggest that chamfer preparations are essential for optimizing the performance of lithium disilicate, as they provide sufficient bulk to support occlusal loads. Zirconia, by comparison, tolerated tangential margins without a significant drop in mechanical strength, supporting its use in minimally invasive preparations. Nevertheless, these conclusions are drawn from controlled in vitro testing and require validation in clinical environments.

The results are consistent with previous reports. Mirkovic et al. [7] and Pan et al. [6] observed that zirconia’s mechanical behavior remains stable across varying margin designs. Similarly, Comlekoglu et al. [1] warned against cervical overcontouring with tangential margins, a risk that our lithium disilicate results support. Additionally, several periodontal studies [10,11] have linked poorly defined margins to an increased inflammatory response, further suggesting that tangential margins may be biologically and biomechanically suboptimal, particularly when used with brittle materials.

Among the strengths of this study are the standardized CAD/CAM [19,20,21] fabrication protocols, controlled cementation methods, and use of validated adhesive systems—10-MDP-based primer for zirconia and hydrofluoric acid/silane for lithium disilicate [22,23,24,25,26,27,28]. These contributed to high internal validity and minimized procedural variability. Load-to-failure testing allowed for a consistent comparison of mechanical performance under compressive stress. Moreover, the adhesive resin cement may have acted as a stress-distributing layer, particularly benefiting the more fracture-sensitive lithium disilicate restorations [27].

Tangential or feather-edge preparations offer clear mechanical advantages in terms of tooth conservation and alignment with minimally invasive dentistry principles [8,17]. They reduce the biological cost of treatment and are particularly beneficial when preserving thin cervical walls or managing deep subgingival lesions [17]. However, these benefits must be weighed against certain clinical challenges. Poorly defined margins can compromise the accuracy of digital impressions [9,28], increase the risk of over-contouring, and lead to plaque retention or soft tissue inflammation, particularly when margins extend subgingivally [2,10,11]. Moreover, the risk of inadequate material thickness may compromise fracture resistance, especially in glass-ceramic restorations [5,6]. As such, the use of tangential margins should be carefully considered based on material selection, periodontal context, and operator proficiency.

From a biological standpoint, margin geometry influences not only the mechanical stability but also the health of surrounding periodontal tissues. Chamfer margins, when executed with precision, offer a well-defined and accessible contour that facilitates accurate impression-taking, optimal marginal adaptation, and effective plaque control. In contrast, tangential or feather-edge margins, while conservative in terms of tooth reduction, may compromise marginal integrity due to their ill-defined termination line, increasing the risk of over-contouring, plaque retention, and soft tissue inflammation [2,10,11]. Furthermore, subgingival placement of indistinct margins can disrupt the supracrestal tissue attachment and exacerbate gingival irritation over time. These factors emphasize the importance of balancing mechanical conservation with biological compatibility when selecting a margin design.

It is also important to note that, despite efforts to standardize tooth preparations through the use of a single experienced operator, clinical reality often involves varying levels of operator skill and technique. Even minor inconsistencies in preparation depth, finish-line location, or taper can alter the geometry and functional outcome of the final restoration. Therefore, while our study minimized procedural variability under in vitro conditions, the reproducibility of such precise preparations in clinical settings may vary, affecting both mechanical performance and long-term biological integration.

Despite its methodological rigor, this study has limitations. The in vitro design lacks simulation of clinical conditions such as thermal cycling, fatigue loading, and biological variability [26,27,28]. Tooth preparations and loading protocols were standardized, which does not reflect the intraoral heterogeneity encountered clinically. Additionally, the absence of fractographic analysis limits insights into failure mechanisms. Future studies incorporating SEM evaluation could help elucidate fracture origin and propagation pathways.

The use of static load-to-failure testing provides controlled conditions for direct comparison but does not replicate the repetitive and variable loading patterns that restorations experience intraorally. Thermo-mechanical aging protocols, including thermal cycling and cyclic fatigue loading, would more accurately simulate long-term functional stresses and should be integrated in future studies to assess restoration durability over time [26,27,28]. Additionally, the absence of fractographic analysis via scanning electron microscopy (SEM) limits our ability to characterize the origin and propagation of cracks. Detailed microscopic evaluation of fracture surfaces could reveal critical failure mechanisms associated with different margin designs and material properties [7,29]. Future investigations incorporating SEM and fatigue-based protocols will be essential to validate and extend these findings under clinically relevant conditions.

To advance clinical relevance, future research should include cyclic fatigue, aging protocols, and clinical trials, ideally using advanced imaging such as micro-CT or optical coherence tomography (OCT) [30,31,32]. Comparative studies across tooth types and margin locations could inform comprehensive guidelines that balance biomechanical strength with biological compatibility. In addition, comparative investigations involving material performance, bonding methods, and prosthetic configurations—including variations in tooth types and margin designs—have been explored in recent studies [33,34,35,36] and could help inform comprehensive guidelines that balance biomechanical strength with biological compatibility.

This study’s limitations include the use of static load-to-failure testing without fatigue aging or thermomechanical simulation, which restricts extrapolation to clinical conditions. Although static testing provides controlled and reproducible measurements, it does not reflect the cyclic and multi-directional forces restorations endure intraorally. Fatigue loading studies have shown that crowns often fail at lower thresholds under repeated stress, particularly in minimally invasive preparations. The absence of a simulated periodontal ligament may have affected stress distribution and fracture behavior [26,27]. Furthermore, fracture mode analysis was limited to visual inspection; the lack of microscopic or SEM evaluation restricts insight into crack initiation and propagation. This study was also confined to mandibular molars, without evaluating the influence of different tooth anatomies, occlusal patterns, or scanner accuracy for various margin designs. Future studies should include fatigue testing, fractographic analysis, and broader clinical parameters to validate and extend these findings [26,27,28,29].

Moreover, the use of a single-point axial load applied via a flat 6 mm indenter does not reproduce the multi-vectorial occlusal forces experienced clinically. Future investigations should consider using more physiologic loading configurations, such as cusp-to-fossa or tripodal schemes, to better approximate intraoral stress distribution. Additionally, the failure mode classification was based solely on macroscopic visual inspection. The absence of microscopic or fractographic evaluation restricts deeper insight into fracture mechanisms. Future studies should incorporate SEM or at least stereomicroscopic analysis to more accurately determine crack initiation points and propagation patterns, enabling a better understanding of material failure behavior.

In summary, zirconia demonstrates high fracture resistance regardless of margin design, supporting its use in conservative preparations. Lithium disilicate, on the other hand, requires well-defined chamfer margins to ensure adequate structural integrity. These findings highlight the importance of material-specific preparation protocols, especially in digitally driven, adhesive prosthodontic workflows.

## 5. Conclusions

Both monolithic zirconia and lithium disilicate crowns exhibited fracture resistance values well above typical masticatory forces, confirming their mechanical suitability for clinical use. However, cervical margin design had a material-specific impact: chamfer margins significantly improved the performance of lithium disilicate crowns, while the multilayer zirconia used in this study (containing both 3Y-TZP and 5Y-TZP) was not significantly affected by margin geometry. These findings suggest that zirconia offers greater flexibility for minimally invasive preparations, whereas lithium disilicate restorations require more robust geometries to optimize structural integrity. The use of CAD/CAM and adhesive workflows enabled consistent fabrication and bonding, reinforcing their value in contemporary prosthodontics. Further in vivo and fatigue-based studies are needed to validate these results under clinically simulated conditions.

## Figures and Tables

**Table 1:** Distribution of fracture modes observed for each material (*n* = 10 per group).

Material	Fracture Mode	Number of Samples	Percentage (%)
Zirconia	Cohesive	7	70%
Zirconia	Catastrophic	3	30%
Zirconia	Adhesive	0	0%
Lithium Disilicate	Cohesive	4	40%
Lithium Disilicate	Catastrophic	6	60%
Lithium Disilicate	Adhesive	0	0%

**Table 2:** Mean fracture load values (N) and standard deviations for each group, with *p*-values from pairwise comparisons.

Restoration Type + Margin Design	Mean (N)	SD (N)	95% CI (N)
Lithium Disilicate–Tangential	1862	251.20	[1715, 2009]
Lithium Disilicate–Chamfer	2494	460.50	[2195, 2793]
Zirconia–Tangential	2425	240.50	[2286, 2564]
Zirconia–Chamfer	2658	541.31	[2329, 2987]

Note: Within the lithium disilicate groups, chamfer vs. tangential difference was significant (*p* < 0.01). Within the zirconia groups, *p* = 0.19 (n.s.). Between materials: significant for tangential margin (*p*<0.01), n.s. for chamfer margin.

**Table 3:** The effects of material type, margin design, and their interaction on fracture resistance. Statistical significance was set at *p* < 0.05.

Source of Variation	F-Value	*p*-Value	Partial η^2^	Significance
Material (Zr vs. LD)	26.47	<0.001	0.49	Yes
Margin Design (Chamfer vs. Tangential)	3.21	0.08	0.07	No
Interaction (Material × Margin)	9.76	<0.01	0.17	Yes

Note: LD = Lithium Disilicate; Zr = Zirconia.

## Data Availability

The original contributions presented in the study are included in the article, further inquiries can be directed to the corresponding authors.

## Abbreviations

The following abbreviations are used in this manuscript:

CAD/CAM	Computer-Aided Design/Computer-Aided Manufacturing
FEA	Finite Element Analysis
LD	Lithium Disilicate
PFM	Porcelain-Fused-to-Metal
SD	Standard Deviation
SEM	Scanning Electron Microscopy
Zr	Zirconia
n.s.	Not Significant
